# TGF-β in tolerance, development and regulation of immunity

**DOI:** 10.1016/j.cellimm.2015.10.006

**Published:** 2016-01

**Authors:** Chris J.C. Johnston, Danielle J. Smyth, David W. Dresser, Rick M. Maizels

**Affiliations:** Institute of Immunology and Infection Research, University of Edinburgh, UK

**Keywords:** Tolerance, Transplantation, Infection, Transforming growth factor

## Abstract

The TGF-β superfamily is an ancient metazoan protein class which cuts across cell and tissue differentiation, developmental biology and immunology. Its many members are regulated at multiple levels from intricate control of gene transcription, post-translational processing and activation, and signaling through overlapping receptor structures and downstream intracellular messengers. We have been interested in TGF-β homologues firstly as key players in the induction of immunological tolerance, the topic so closely associated with Ray Owen. Secondly, our interests in how parasites may manipulate the immune system of their host has also brought us to study the TGF-β pathway in infections with longlived, essentially tolerogenic, helminth parasites. Finally, within the spectrum of mammalian TGF-β proteins is an exquisitely tightly-regulated gene, anti-Müllerian hormone (AMH), whose role in sex determination underpins the phenotype of freemartin calves that formed the focus of Ray’s seminal work on immunological tolerance.

## Highlights

• The broader superfamily of TGF-β-like proteins is reviewed, and signaling pathways summarised.
• The role of TGF-β in the immune tolerance and control of infectious disease is discussed.
• The superfamily member AMH is involved in embryonic sexual differentiation.
• Helminth parasites appear to exploit the TGF-β pathway to suppress host immunity.
• TGF-β homologues and mimics from parasites offer a new route for therapeutic tolerance induction.

## Graphical abstract

**Figure:**
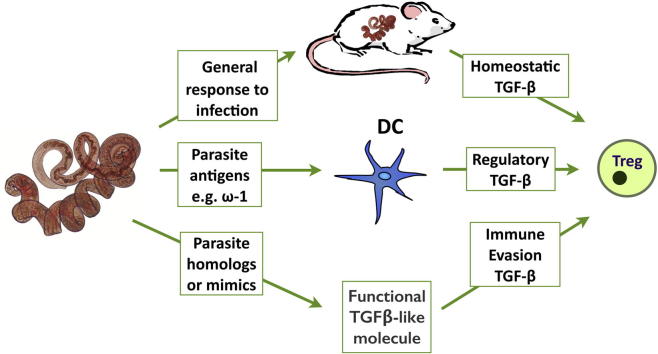

## 1 Introduction

The TGF-β superfamily is an ancient metazoan protein class which cuts across cell and tissue differentiation, developmental biology and immunology. Its many members are regulated at multiple levels from intricate control of gene transcription, post-translational processing and activation, and signaling through overlapping receptor structures and downstream intracellular messengers. We have been interested in TGF-β homologues firstly as key players in the induction of immunological tolerance, the topic so closely associated with Ray Owen. Secondly, our interests in how parasites may manipulate the immune system of their host has also brought us to study the TGF-β pathway in infections with long-lived, essentially tolerogenic, helminth parasites. Finally, within the spectrum of mammalian TGF-β proteins is an exquisitely tightly-regulated gene, anti-Müllerian hormone (AMH), whose role in sex determination underpins the phenotype of freemartin calves that formed the focus of Ray’s seminal work on immunological tolerance.

## 2 The TGF-β superfamily

TGF-β was named for its ability to drive fibroblast proliferation before its broader role in development and immunity had been established; in the meantime related proteins, such as the Bone Morphogenetic Proteins (BMPs) were first characterized in vertebrates before becoming discovered in *Drosophila* flies, *Caenorhabditis elegans* nematodes and other lower animals. Even within the vertebrates, there are more than 30 distinct molecules including three isoforms of TGF-β, Bone Morphogenetic Proteins (BMPs), activins, inhibins, nodal and growth differentiation factors, and anti-Müllerian hormone (AMH) [1], [2], [3], [4]. Structurally, these proteins are synthesized as ∼400-amino acid inactive pre-proteins, and are cleaved by furin-like proteases to yield an active ∼110-amino acid C-terminal domain; the active domain is tightly cross-linked with 3–4 disulfide bonds, and generally forms a homodimer through one additional cysteine residue on each chain.

The ligand family is complemented by a wide-ranging set of receptors, which canonically are heterodimers composed of two serine-threonine receptor kinases, termed Type I and Type II [3]. Depending on the TGF-β family member and expression of appropriate receptors on the surface of cells, these ligands initiate pleiotropic effects in a broad spectrum of biological processes including embryogenesis, immunity, angiogenesis and wound healing [5]. Reflecting this remarkable multiplicity of events dependent on closely related TGF-β proteins, a complex and highly-regulated signaling arrangement exists [6], [7].

Regulation of TGF-β signaling takes place in three distinct settings: the extracellular space, the cell membrane and the intracellular region. The full-length TGF-β pro-protein is cleaved to produce not only the *C*-terminal homology domain, but also a functional *N*-terminal ‘latency-associated peptide’ (LAP) which remains non-covalently attached to the cytokine polypeptide thereby retaining it in inactive form. Prior to secretion from a cell, the LAP-TGF-β complex binds to a further protein, latent TGF-β binding protein (LTBP). Because TGF-β is secreted in this way, as a component of a biologically inactive compound, processes that liberate the active TGF-β molecule can be as important to immunomodulation as its transcription and synthesis [8]. In the extracellular space, the active TGF-β molecule is also prone to sequestration by ‘ligand trap’ proteins (including LAP), which limit the duration and range of active TGF-β stimulation [9].

*In vivo*, cell surface receptors including many integrins have the ability to bind LAP-TGF-β and release active TGF-β [10]. This mechanism is of considerable importance for example αv integrin null mice develop similar embryological aberrations to TGF-β^−/−^ animals [11]. Furthermore, even if integrin-TGF-β interaction is ablated solely within the dendritic cell compartment, the resultant immunological dysfunction is sufficient to induce spontaneous colitis in normal environmental conditions [12].

**Fig. 1:**
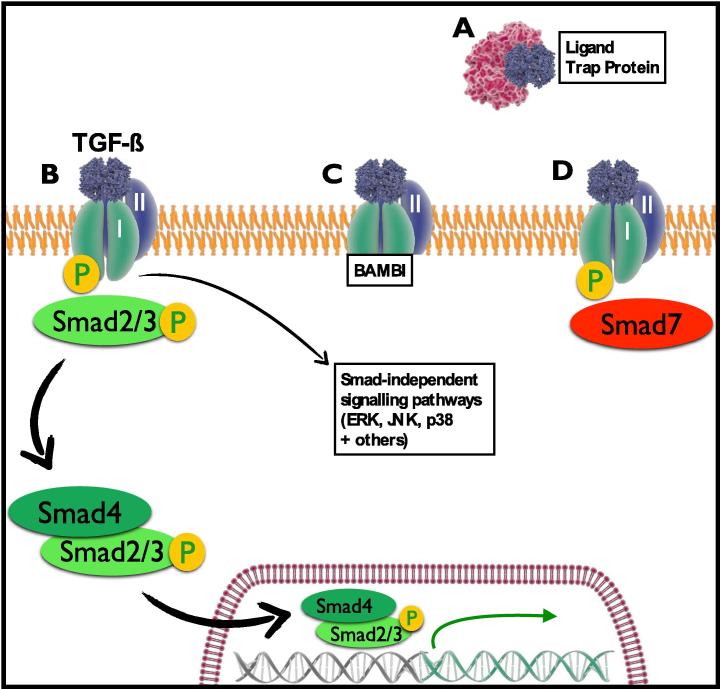
TGF-β signaling and regulation. (A) Active TGF-β is prevented from binding to receptors due to incorporation with ‘ligand trap proteins’ such as latency-associated peptide (LAP). (B) Binding of TGF-β to the Type II TGF-β receptor leads to phosphorylation of the Type I TGF-β receptor intracellular domain and activation of the Smad signaling pathway, in addition to Smad-independent signaling including MAPK pathways. (C) Decoy receptors such as BAMBI bind TGF-β but prevent downstream signaling. (D) Smad7, an inhibitory Smad, binds to the phosphorylated Type I TGF-β receptor and prevents downstream signaling.

Activated TGF-β signals as a homodimer via the union of two type I TGF-β receptors and two type II TGF-β receptors. In humans, 5 variants of the type I receptor and 7 variants of the type II receptor have been identified, in contrast to 29 potential ligands [13]. The affinity of each ligand for individual receptors varies, but most ligands are also able to bind multiple heteromeric combinations of type I and type II receptors, resulting in different downstream effects [13]. Upon binding of the TGF-β ligand, the constitutively active type II receptor is brought into close proximity of the type I receptor, thereby enabling phosphorylation of the TβRI intracellular ‘GS’ domain and initiating the Smad signaling cascade [14] (Fig. 1).

Smads are intracellular proteins first identified in *C. elegans* worms (as *Sma* genes in small phenotype organisms [15]) and *Drosophilia* flies (as *Mad* genes [16]) and are the key intermediaries in signaling from TGF-β receptors to the nucleus. Hence, not only the ligands but the entire signaling pathway is conserved in the animal kingdom, including parasites such as *Schistosoma mansoni* [17], [18].

Eight Smad proteins have been identified in vertebrates and they are sub-categorized according to their function: receptor-activated Smads (R-Smads), common Smads (Co-Smads) and inhibitory Smads (i-Smads) [14]. When TGF-β binds and activates the TGF-β receptor complex, the intracellular GS domain of TβRI phosphorylates Smad2 and Smad3 (R-Smads), which then form a complex with Smad4 (Co-Smad) and enter the nucleus to initiate gene transcription [19]. This process can be regulated intracellularly by Smad7, an inhibitory Smad that can bind TβRI, prevent further signal transduction and then stimulate proteolytic degradation of the receptor [14]. The importance of the i-Smad7 is underlined by observations that it is upregulated in inflammatory bowel diseases, and colitis in mice can be attenuated by antisense Smad7 oligonucleotides [20]. IRF3 is a related transcription factor which can bind and inactivate Smad3; this mechanism may be important in viral infections to suspend TGF-β-mediated immunoregulation until the pathogen is cleared [21].

At the level of the cell membrane, another regulatory mechanism comes into play in the form of ‘decoy’ receptors such as BAMBI (BMP and activin membrane-bound inhibitor), a transmembrane protein that is structurally very similar to TβRI, but lacks an intracellular GS domain [22]. BAMBI can therefore form a dimer with TβRII and bind TGF-β without initiating any Smad signaling and thereby reduce the number of TβRIIs available to bind other TGF-β molecules.

The Smad cascade is the ‘canonical’ signaling pathway for TGF-β and is essential for TGF-β-driven immunoregulation and Treg/Th cellular differentiation [23]. However, TGF-β is also able to activate a number of Smad-independent signaling pathways including mitogen-activated protein kinases (MAPKs). Of these, ERK phosphorylation is an important event in the process of epithelial to mesenchymal transition (EMT), which is necessary in embryological development, but can contribute to pathological fibrosis, one of the major drawbacks in current strategies for therapeutic applications of TGF-β [24].

## 3 TGF-β in the immune system

TGF-β is a broadly immune suppressive mediator which can, for example, block allergic inflammation in the lung [25] and autoimmune diabetes in the pancreas [26]. Deficiency in either the cytokine or its receptors results in fulminant inflammatory disease that proves lethal in the first weeks of life [27], a phenotype that can be reproduced even if only T cells are unable to respond to TGF-β [28]. The cytokine is instrumental in almost every compartment of the immune system [29], inducing for example B cell class switching to IgA [30], [31] and driving myeloid cells into a more tumor-promoting phenotype [32]. But its effects on T cells are perhaps the most prominent, in particular its ability to stimulate naïve CD4^+^ T cells to differentiate into Foxp3^+^ Treg that can suppress effector T cell activation and proliferation [33], [34], and prolong allograft survival upon adoptive transfer into recipient animals [35]. The level of Foxp3 expression by Treg correlates with functional suppressive capacity [36] and stability of Foxp3 expression is essential for maintenance of a regulatory phenotype [37]. Additionally, TGF-β is capable of promoting a tolerant state through Foxp3-independent mechanisms, such as upregulation of CD73 [38], an ectoenzyme that acts to increase the local extracellular concentration of adenosine.

The combination of TGF-β and certain other cytokines, may induce T cells to differentiate into non-regulatory phenotyopes such as Th17 effectors in the presence of IL-6 [39] and Th9 when IL-4 is present [40]. The action of TGF-β on T cells is antagonized by IFN-γ and IL-4, representing an important pathway by which these cytokines suppress Th17 differentiation [39].

## 4 TGF-β and regulatory T cells

CD4^+^ regulatory T cells (Treg), identified by expression of the transcription factor Foxp3, are arguably the single most important cell type in mediating peripheral tolerance [41], [42]. Regulatory T cells are subdivided into two types [43]. Thymic Treg (tTreg) constitutively express Foxp3 before leaving the thymus, independently of TGF-β, and play the predominant role of maintaining tolerance to self antigens. In contrast, peripherally-induced Treg (pTreg) develop from naïve, mature CD4^+^ cells exposed to antigens under tolerogenic conditions (for example by immature DCs with low levels of co-stimulation), and with an essential requirement for TGF-β signaling [44].

Tregs are crucial for physiological immune homeostasis and their absence leads to severe autoimmunity, which is universally fatal in ‘scurfy’ mice that lack Foxp3 expression [45] and manifests as a life-limiting multisystem disorder in humans – the immune dysregulation, polyendocrinopathy, enteropathy, X-linked syndrome (IPEX) [46], [47]. However, Tregs do also have the potential to cause harm by permitting neoplastic cells to evade anti-tumor immunity [48] and preventing immunity in infections [49]; the long term effects of artificially manipulating Treg populations *in vivo* are therefore unknown.

Other subsets of T cells exert regulatory effects while not expressing Foxp3, through the release of other suppressive cytokines, in particular IL-10 and IL-35 from Tr1 [50] and iTr35 [51] cells. While these have potent down-modulatory functions, TGF-β is unique in being central to both the induction and function of Tregs.

## 5 TGF-β in transplantation

With the potential to synergise with pro-inflammatory cytokines such as IL-6, TGF-β has the potential to play janiform roles in the context of transplantation [52]. In many experimental animal models, TGF-β-induced immunoregulation, and regulatory T cells in particular, clearly promote tolerogenesis and allograft survival. However, detrimental effects can also arise through interstitial fibrosis as a result of increased myofibroblast differentiation; in addition, the ability of TGF-β to promote Th17 differentiation in the presence of pro-inflammatory cytokines (IL-1β, IL-6) and TLR ligands poses a major threat to transplant acceptance [53]. This clearly cautions against the use of TGF-β activity as a strategy to improve graft survival. However, in the absence of TGF-β, Th17 cells can still develop and indeed are more pathogenic due to enhanced IL-23 receptor expression [54]. Furthermore, higher concentrations of TGF-β are able to overcome the Th17 pathway and restore Treg differentiation [55], arguing that, depending on dose and context, TGF-β may still provide a therapeutic option.

## 6 TGF-β in infectious diseases

The central immunosuppressive role of TGF-β is also reflected in many infectious diseases, particularly in chronic infections. Helminth parasites, which typically establish themselves as long-term residents in the mammalian host are often associated with both generalized immunosuppression and elevated TGF-β expression [56], [57]. Moreover, patients with onchocerciasis [58] and lymphatic filariasis [59] show *in vitro* parasite antigen-specific T cell hyporesponsiveness which can be reversed with anti-TGF-β antibodies. In human helminth infections, IL-10 is also a very prominent immunosuppressive factor [60]; whether this is driven by high levels of TGF-β remains to be ascertained.

Alongside the elevation of TGF-β cytokine, many infectious disease settings are accompanied by expansion of Foxp3^+^ Tregs [49]. Specifically in the context of helminth parasitism, in murine infections with *Brugia malayi* [61], *Heligmosomoides polygyrus* [62], [63], [64] and *Strongyloides ratti* [65] all drive marked increases in Tregs, which in the latter two models can be shown to functionally inhibit the host Th2 protective response and promote chronic infection; moreover, blocking TGF-β signaling in *H. polygyrus* infection results in greater worm expulsion [66], establishing a mechanistic link to this key cytokine. The activity of Tregs is also enhanced in many human helminth infections and, together with other regulatory pathways, may establish a form of immunological tolerance to parasites allowing them to remain in the body for many months or years [67], [68].

**Fig. 2:**
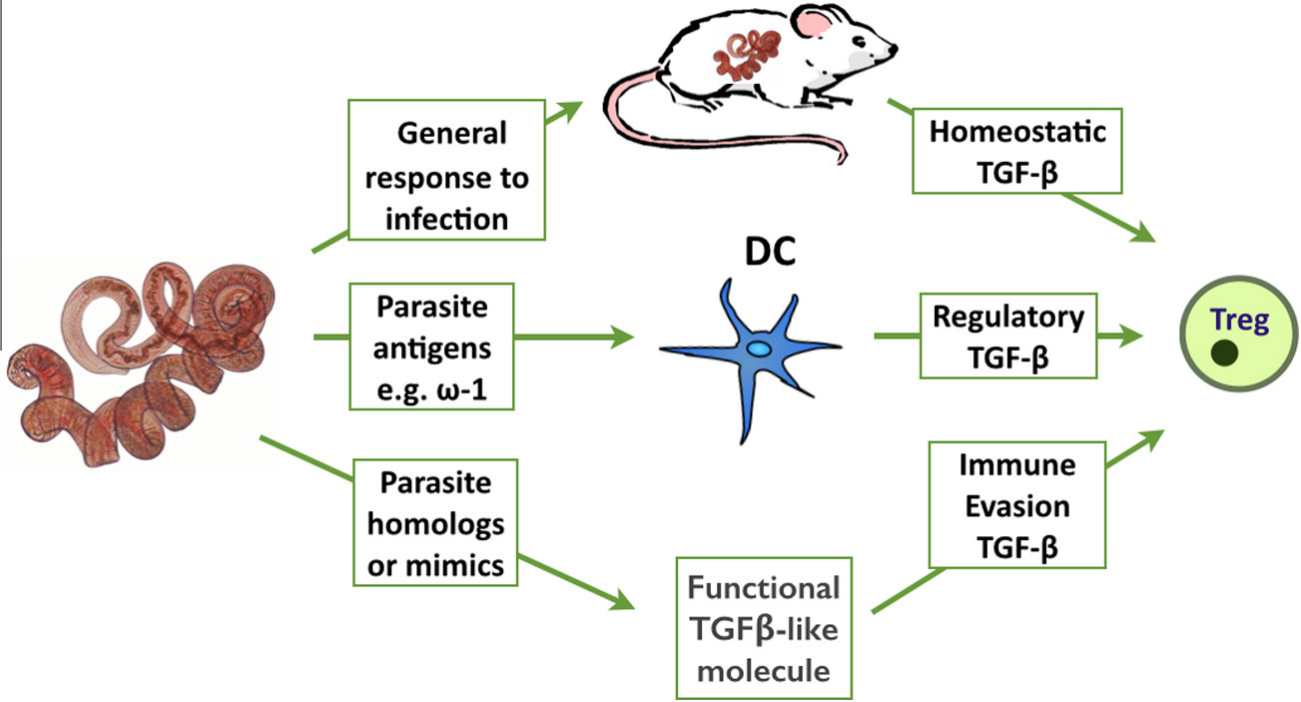
Helminths and the TGF-β pathway. Several mechanisms may operate to raise TGF-β levels in parasite infection, such as (i) host homeostasis to minimize immunopathology in chronic infection; (ii) pathogen triggering of TGF-β production or activation by host cells such as DCs; or (iii) parasite expression of homologues or mimics of the host cytokine to drive the same pathway as host TGF-β.

Several mechanisms may operate to raise TGF-β levels in parasite infection, such as (i) host homeostasis to minimize immunopathology in chronic infection; (ii) pathogen triggering of TGF-β production or activation; or (iii) parasite mimicry of the host cytokine to drive the same pathway as host TGF-β. In fact, examples of all 3 can now be found (Fig. 2).

## 7 Host homeostasis or pathogen ploy?

Every immune response must engender a regulatory component that will eventually dampen and terminate that response [69]; hence an increase in TGF-β and Tregs during an infectious episode may reflect host homeostatic mechanisms rather than a strategy evolved by pathogens to suppress immunity.

In a number of parasite models, however, interfering with the TGF-β pathway results in greater resistance to infection; although inferential, these findings are often taken to argue that pathogens benefit from (and may therefore have evolved to favor) TGF-β signaling. These include *in vivo* studies with SB431542, an inhibitor of the ALK5 receptor kinase, [66], and *in vivo* antibody neutralization of host TGF-β [70], each of which lead to greater expulsion of the chronic gastrointestinal parasite *H. polygyrus*.

Interestingly, mice in which T cells express a dominant negative TGF-βRII are not more resistant to helminth infection, but show marked overexpression of inflammatory cytokines such as IFN-γ, blocking an effective type 2 anti-parasite response [71], [72]; in parallel, induction of IL-10 is abrogated in infected mice lacking T cell TGF-β signaling, emphasizing the importance of TGF-β in driving other pathways of immunosuppression [71]. Deletion of the TGF-βRII only in myeloid cells, however, results in enhanced immunity to intestinal helminths, indicating that the cytokine may normally inhibit a protective effector myeloid phenotype *in vivo* [73].

Furthermore, while TGF-β levels are elevated in many helminth infections, clearance of parasites through anthelmintic chemotherapy reduces cytokine levels, indicating that the helminths may be actively inducing high expression [74]. Conversely, in the *B. malayi* model system, Foxp3^+^ Treg expansion required live parasite infection [61]; the inability of dead parasites to induce a regulatory response indicated that Treg expansion is driven by the activity and/or products of live helminths. In some cases, this process may be indirect, as for example in the case of ES of *Trichinella spiralis*, which stimulates host DCs to induce Tregs [75].

## 8 Parasite induction or activation of host TGF-β

TGF-β levels increase in many different helminth infections, although only in some instances has a causal mechanism been established by which parasites initiate a pathway to ensure cytokine production. For example, in infection with the helminth *S. mansoni*, a regulatory cascade is generated through soluble egg antigens (SEA) that induce T cell differentiation into Foxp3^+^ Tregs [76]; the effect is sufficient to protect diabetes prone NOD mice from developing autoimmunity. SEA is also capable of suppressing CD4^+^ T cell proliferation and inducing Foxp3 expression indirectly. Whist SEA-driven Foxp3 induction has only been demonstrated in the presence of DCs (and not culture with CD4^+^ T cells alone), an increase in the expression of TGF-β latency associated peptide on the surface of CD4^+^ cells suggests that SEA also induces enhanced secretion of TGF-β by activated T cells, further facilitating Foxp3^+^ Treg differentiation [77]. In humans, although the mechanism has yet to be defined, helminth infections such as *Onchocerca volvulus* elicit extremely high local levels of TGF-β from many cell types around the nodules in which adult parasite establish themselves [78].

Pathogens may also directly promote activation of TGF-β, as was found in the case of influenza A neuraminidase activating the latent TGF-β complex in epithelial cells and tissues [79]; furthermore, the consequent upregulation of extracellular matrix proteins facilitated the adhesion and infection with streptococcal bacteria, thereby promoting secondary infection of the host.

## 9 Parasite-derived ligands – homology or mimicry?

We postulated that helminths may express TGF-β homologues that could interfere with host immunity, and characterized several members of the gene superfamily listed in Table 1 along with those identified by other laboratories. In particular, we characterized two ligands in *Brugia* [80], [81] and four in *H. polygyrus* [82]. One, *Bm*-TGH-2 from *B. malayi*, was found to activate the MFB-F11 reporter cell line [81], but in unpublished work we found the closest homologue from *H. polygyrus* did not do so. In *Onchocerca*, antibody to mammalian LAP revealed widespread staining of somatic tissues, indicating a parasite-encoded protein sufficiently similar to LAP/TGF-β to be recognized by antibodies [83]. In platyhelminths (flatworms) activin-like homologues have been identified in both *S. mansoni* [84], [85] and *Echinococcus multilocularis* [86]; since mammalian Activin A can also stimulate Foxp3 induction and Treg development [87], [88], it will be interesting to learn if these parasite ligands can act likewise.

Despite the inability of *H. polygyrus* TGF-β homologues to drive signaling, the proteins secreted by this parasite (termed HES) did directly induce Foxp3^+^ Treg differentiation in isolated CD4^+^Foxp3(GFP)^−^ T cells *in vitro* with stimulation from Concanavalin A or plate-bound CD3/CD28 [66]. HES also suppressed proliferation of CD4^+^Foxp3(GFP)^−^ T cells and promoted IL-17 expression from naïve CD4^+^ cells when co-cultured with IL-6. This led to further investigation with a TGF-β reporter cell line (TGF-β^−/−^ fibroblasts transfected with a TGF-β-responsive alkaline phosphatase reporter), which confirmed TGF-β activity within HES that could be completely ablated with a type I TGF-β receptor kinase inhibitor, but was unaffected by a pan-vertebrate anti-TGF-β blocking antibody. Thus, HES contains a TGF-β mimic that induces Foxp3 through ligation of the TGF-β receptor complex, but is sufficiently structurally dissimilar to mammalian TGF-β as to be unaffected by a neutralizing antibody. Recently, in work to be published elsewhere, we have isolated the gene encoding a novel protein with no sequence similarity to the TGF-β family, which is able to ligate the mammalian receptor.

Similar expression of Foxp3 in T cells has been reported to be induced by secreted proteins from some other helminths, such as the fox tapeworm *E. multilocularis* [89], and the ruminant nematode *Teladorsagia circumcincta* [66]. Most recently, products from the *Litomosoides sigmodontis* filarial parasite have been shown to ligate the host TGF-β receptor [90]. In each case, the active principles have yet to be determined.

## 10 TGF-β homologues in helminth arrested development

Originally the TGF-β superfamily gene *daf-7* was found to be a key player in controlling entry into the arrested larval stage of *C. elegans*, the Dauer larvae, which follows the loss of *daf-7* expression [91]; in this model, *daf-7* null mutants constitutively entered Dauer arrest. Parasitic helminths enter crucial and often long-lasting developmental arrest, for example as infective larvae awaiting the opportunity to enter a new host, it was plausible that TGF-β family members might similarly regulate arrest in parasites [80], [92]. However, contrary to this prediction, in a number of parasitic species in which TGF-β superfamily homologues were discovered (Table 1), expression was found to be maximal in the arrested third larval instar (L3) stage that is most closely analagous to the *C. elegans* Dauer larva [93], [94], [95], [96], [97]. This suggests that either that there has been a functional reversal in the TGF-β signaling pathway between free-living and parasitic nematodes, or that this cascade is not critical to the developmental program of the parasites examined. In a further departure from expectation, the closest *daf-7* homologue in *B. malayi*, TGH-2, is highly expressed in the newborn L1 stage, the microfilaria, which enters arrest in the bloodstream of the host until uptake by hematophagous mosquitos [81]. Whether this reflects an unusual plasticity in the role of TGF-β ligands in nematode development has yet to be investigated.

In platyhelminths, the developmental role of superfamily members is also being analyzed [18], [84], [98]. However, recent work has screened genomic DNA sequences in the liver fluke *Fasciola hepatica*, identifying 3 homologues, one of which (FhTLM) is able to enhance egg embryogenesis and motility of juvenile parasites when administered as a recombinant protein [99]. Further homologs of the TGF-β receptor superfamily and Smad signaling proteins have also been characterized from several major parasitic helminth species (Table 2).

## 11 TGF-β homologue in embryonic sexual differentiation

During early development anti-Müllerian hormone (AMH) ablates the Mullerian duct (the precursor of the oviduct) in the male mammal; the only cells expressing AMH are the Sertoli cells which generate high levels of secreted hormone in the developing organism – at 11.5 to 12.5 days post-conception in the embryonic mouse. Amh is secreted at lower levels in the male until puberty and in the female by granulosa cells after puberty. Regulation of AMH is one of the most rigorous examples of control in the genome, and is initiated following expression of the Sry sex determining region on the Y chromosome [100]. The *amh* locus is highly conserved on human chromosome 19 and mouse chromosome 10, adjacent to widely expressed housekeeping genes; hence the *amh* promoter appears to be tightly constrained within a few hundred nucleotides of the start site [101].

Working with the murine Sertoli cell line SMAT-1, expression of AMH was also found to require an enhancer immediately downstream of the 3′ polyadenylation site. At the promoter level it has been found that expression is extremely finely regulated by enhancing (eg GATA1) and inhibitory (eg GATA4) transcription factors binding to noncoding regions (elements) of the gene which are highly conserved between mammalian species [102], [103], [104]. In particular, a high level of expression requires the presence of an enhancer motif immediately downstream of the 3′ polyadenylation site [105]. Mutation of an element within this motif, or of the Wilms tumor element which lies upstream of the gene, ablate high level expression of *amh*; these sites can be considered as anchor points for a specific bridging factor. Remarkably, mutation of a site lying a few nucleotides upstream of the enhancer anchor point leads to an accentuated *amh* expression. Looping between motifs on either side of the coding sequence is necessary for strong activation of the gene.

Interestingly, if cattle conceive a heterosexual pair of twins, placental anastomoses expose the female fetus to the inhibitory effects of AMH in utero, resulting in a masculinized infertile individual known as a freemartin [106]. The other consequence of the anastomosis is hematological chimerism, resulting in establishment of immunological tolerance between the twins, as reported in Ray Owen’s landmark study in 1945 [107].

## 12 New therapeutic strategies?

The potency and breadth of effect of TGF-β ligands suggest many therapeutic scenarios to treat inflammatory diseases and facilitate transplantation. While the non-linear signaling and pleiotropic activities of TGF-β present significant therapeutic challenges, a considerable unmet clinical need currently exists across many severe conditions, and recent advances in understanding have brought the goal of driving immunological tolerance several steps closer. The approaches currently being examined include direct application to dampen inflammation, administration *in vivo* to induce tolerance, and use *ex vivo* to condition patients’ T cells into the regulatory phenotype.

Direct administration is currently the least favored strategy, in part because of the pro-fibrotic role of TGF-β which efforts to date have not well dissociated from its immunosuppressive role. There are also concerns that generalized immune suppression resulting from administration of TGF-β might present risks of infection or neoplasia comparable to those of current non-specific immunosuppression regimens.

In recent years, attention has been drawn to the possibility of administering live helminth infections to attenuate or pre-empt inflammatory disorders [108]. From a safety perspective, treatment with low doses of helminth infection may not be hazardous, judging by the millions of people chronically infected with helminths worldwide, of whom very few experience immunological sequelae that approach those of current routine immunosuppression therapy [109], [110]. However, the balance between therapeutic efficacy and parasite pathogenicity is not well understood, and is likely to depend not only upon the parasite species in question, but also the genetic predisposition of the host [111], so that adverse effects in a minority of recipients cannot be excluded. Nevertheless, a total of 28 clinical trials of therapeutic helminth infection are now underway or have been completed [112]. While adverse effects do appear to be reassuringly limited, the proposal of experimentally infecting patients with live helminths still engenders a wide range of regulatory, logistical and scientific challenges, such that its unequivocal validation as a beneficial and viable therapy remains elusive [113].

Identification and reproduction of individual helminth-secreted immunomodulatory molecules as potential novel therapeutic agents presents several advantages over live larval therapy [114]. These include consistent pharmacokinetics, scope for pharmacological modification and optimization (reducing immunogenicity of large molecules, for example), improved public acceptability and a lower cost barrier to large-scale production as a routine clinical therapy. Compared with recombinant human TGF-β, it is also likely that helminth-derived homologues have evolved to evade some mechanisms of endogenous TGF-β regulation and may therefore provide the opportunity for greater precision in dosing and specificity of action.

Combination therapies are another approach in development, whereby TGF-β is administered with synergistic ‘Treg permissive’ agents which may allow some control over downstream cellular differentiation. Of these, rapamycin (sirolimus) can act synergistically with TGF-β to favor Foxp3 expression and Treg differentiation over Th17 effector cells [115], while retinoic acid (RA) is thought to minimize the impact of inflammatory cytokines and co-stimulation on impairing TGF-β-induced Foxp3 expression [116], [117].

TGF-β-dependent induction of Treg also occurs *in vivo* and techniques that exploit this mechanism to induce tolerance (such as low dose antigen therapy [118]) appear to generate Treg with more stable expression of Foxp3 than those generated *in vitro* [119]. This suggests that additional stabilizing factors or conditions are present in the *in vivo* setting and might provide encouragement for *in vivo* Treg induction techniques over *ex vivo* expansion and reinfusion of isogeneic cells.

Nevertheless, prevailing concerns about *in vivo* administration of TGF-β are encouraging emphasis on *in vitro* use to generate Tregs which are then administered to patients. In mouse models, adoptively transferred Treg mediate indefinite tolerance of murine allografts including pancreatic islet [120], skin [121] and heart [122]. Expectations of successful translation of Treg therapy into the clinical setting have been high and preliminary clinical trials have now been completed in graft-versus-host disease [123] and hematopoietic stem cell transplantation [124] with modest but encouraging results. To ensure that transfused Tregs are specific for the pathogenic epitope (such as an auto- or allo-antigen), it is also possible to transduce patient T cells *in vitro* with an engineered T cell receptor, creating a highly-targeted and effective regulatory T cell population [125].

However, a number of obstacles and concerns persist. First, Good Manufacturing Practice (GMP)-compliant *ex vivo* expansion of Tregs for subsequent reinfusion is a highly specialized process at a cost of approximately $45,000 per patient [126]. Even if this level of funding could be justified, the infrastructure and highly qualified personnel required are likely to limit translation into routine clinical practice.

Secondly, due to the lack of a unique human regulatory T cell surface marker, accurate identification of Treg populations remains imperfect. The optimal approach is with fluorescence-activated cell sorting (FACS), with selection of, for example, CD4^+^CD25^+^CD127^lo^ cells [124]. However, GMP-compliant FACS isolation of Treg for clinical therapeutic use is available at very few centers throughout the world necessitating the use of magnetic cell sorting techniques for preliminary clinical trials, with Treg populations consequently of a lower purity [127], [128]. Additional steps such as CD8^+^ T cell depletion can limit alloreactive effector T cells to a very small percentage, but it is likely that any remaining are highly activated, and the long-term impact of their infusion into a transplant recipient is unpredictable [128].

Thirdly, concern remains over the question of whether isolated Treg maintain their regulatory phenotype following re-infusion, particularly in the context of an inflammatory environment. Alloantigen-specific pTreg offer the potential advantages of high functional suppressive ability and a specificity of action that might lower the risk of side effects such as early viral reactivation (observed in trial of Treg therapy in hematopoietic stem cell transplantation [129]) and the potential risk of neoplasia with non-specific Treg therapy. A further caution has been the loss of Foxp3 expression (and therefore regulatory phenotype) once induced Tregs are no longer exposed to TGF-β [130]. This poses a risk of infusing a population of cells that effectively revert to allograft-specific effector T cells, and the ability or otherwise to treat this scenario with conventional immunosuppression is unknown [131]. Therapeutic infusion of tTreg and pTreg comprise two separate arms of the ONE Study that is currently underway (NCT02129881).

Finally, in the long-term it is unknown whether Treg-mediated immunosuppression might present risks of infection or neoplasia comparable to those of current non-specific immunosuppression regimens. To date, four clinical trials of Treg therapy have been published: three investigating prevention or treatment of graft vs host disease (GvHD) [123], [132], [133] and one for treatment of type I diabetes [134]. Early follow-up has provided some degree of reassurance, with no adverse events reported other than a slightly increased incidence of viral reactivation in the context of GvHD [129]. However the longest follow-up period that has been reported is only 12 months [134] and, particularly regarding a potential long-term risk of malignancy, it may be very difficult to determine a follow-up period wherein this question can be answered definitively. In short, Treg cellular therapy is an attractive potential therapeutic strategy that has advanced rapidly in recent years, but many questions and logistical barriers still exist, such that translation to routine clinical practice is by no means guaranteed.

## Figures and Tables

**Table 1:** TGF-β ligands in helminth parasites.

Species	Ligands	Properties	References
Ancylostoma caninum	Dbl-1 like, Daf-7 like (TGH-2)	Upregulated in arrested L3 larvae	[93], [95]
Brugia malayi	Bm-TGH-1, TGH-2	TGH-2 ligated TGF-β reporter cell line	[80], [81]
Echinococcus multilocularis	Activin homolog	Induces Foxp3	[86]
Fasciola hepatica	Fh-TLM and 2 other homologs	Fh-TLM promotes development	[99]
Haemonchus contortus	Hc-TGH-2	Upregulated in L3 larvae	[82]
Heligmosomoides polygyrus	Hp-TGH-2	Upregulated in adult worms and eggs	[82]
Nippostrongylus brasiliensis	Nb-TGH-2	Upregulated in L3 larvae	[82]
Parastrongyloides trichosuri	Daf-7 like	Upregulated in L3 larvae	[94]
Schistosoma japonicum	SjBMP	Ovarian and tegumental expression	[135]
Schistosoma mansoni	SmInAct	Functions in embryogenesis	[84], [85]
Strongyloides ratti	Daf-7 like	Upregulated in L3 larvae	[94]
Strongyloides stercoralis	Sst-TGH-1		[96]
Teladorsagia circumcincta	Tci-TGH-1; TGH-2		[96], [82]

**Table 2:** TGF-β family receptors and Smad signaling proteins in helminths.

Species	Receptors	Properties	References
Brugia malayi and Brugia pahangi	Bm-TGR-1, -2		[136]
Echinococcus multilocularis	EnTR1	Interacts with host BMP2	[137]
Schistosoma mansoni	RI + RII	Interact with host TGFβ	[138], [139], [140]

Species	Smad signaling proteins	Properties	References

Echinococcus multilocularis	EmSmadA-D	A and C lack MH1 domain	[141], [142]
Echinococcus multilocularis	EmSmadE	Phosphorylated by human BMP and TGFβRI	[143]
Schistosoma mansoni	Smad proteins		[144], [145]
